# The Self‐Reference Effect on *Perception*: Undiminished in Adults with Autism and No Relation to Autism Traits

**DOI:** 10.1002/aur.1891

**Published:** 2017-11-21

**Authors:** David M. Williams, Toby Nicholson, Catherine Grainger

**Affiliations:** ^1^ University of Kent Canterbury UK; ^2^ University of Stirling Stirling UK

**Keywords:** autism spectrum disorder, self‐reference effect, self‐awareness, metacognition, mindreading, memory, perception

## Abstract

Memory for (and perception of) information about the self is superior to memory for (and perception of) other kinds of information. This self‐reference effect (SRE) in *memory* appears diminished in ASD and related to the number of ASD traits manifested by neurotypical individuals (fewer traits = larger SRE). Here, we report the first experiments exploring the relation between ASD and the SRE in *perception*. Using a “Shapes” Task (Sui et al., Journal of Experimental Psychology: Human Perception and Performance, 38, 1105, 2012), participants learned to associate three different shapes (triangle, circle, square) with three different labels representing self, a familiar other, or an unfamiliar other (e.g., “you”, “mother”, “stranger”). Participants then completed trials during which they were presented with one shape and one label for 100 ms, and made judgments about whether the shape and label was a match. In Experiment 1, neurotypical participants (*n* = 124) showed the expected SRE, detecting self‐related matches more reliably and quickly than matches involving familiar or unfamiliar other. Most important, number of ASD traits was unrelated to the size of the SRE for either accuracy or RT. Bayesian association analyses strongly supported the null hypothesis. In Experiment 2, there were no differences between 22 adults with ASD and 21 matched comparison adults in performance on the Shapes Task. Despite showing large and significant theory of mind impairments, participants with ASD showed the typical SRE and there were no associations with ASD traits in either group. In every case, Bayesian analyses favored the null hypothesis. These findings challenge theories about self‐representation in ASD, as discussed in the article. ***Autism Res*** *2018, 11: 331–341*. © 2017 The Authors Autism Research published by International Society for Autism Research and Wiley Periodicals, Inc.

**Lay Summary:**

Neurotypical people tend to find it easier to perceive and remember information that relates to themselves than information that relates to others. Research suggests that people with ASD show a diminished (or absent) self‐bias in *memory* and that severity of ASD predicts the extent of this diminution (more severe ASD = smaller self‐bias in memory). However, the current research suggests strongly that people with ASD do show a self‐bias in their perception. This research informs our understanding of psychological functioning in ASD and challenges theories regarding self‐awareness in this disorder.

## Introduction

Definitions of self‐awareness are numerous, throughout the history of philosophy as well as psychology. One particularly important distinction between subjective and objective levels of self (the ‘I’ and ‘me’, respectively) was drawn by James [1890]. On the one hand, the self is an existential entity that experiences (the “I”). It is the knower, the experiencer, and the agent of activity. On the other hand, the self can be both know*n* and experienc*ed* (the “Me”). The self can be the object of thought.

Regardless of the precise taxonomy of self that is employed, it is widely agreed that the self plays an important role in human cognition and perception, exerting influence across a range of domains and situations [see Sui & Humphreys, 2015]. One of the clearest empirical demonstrations of this influence is the so‐called “self‐reference effect” [Rogers, Kuiper, & Kirker, 1977], whereby information encoded in relation to the self has a mnemonic advantage over information encoded in other ways. This effect is apparent in a number of different paradigms and across different domains of processing. For example, in the domain of memory, when people are asked to make explicit yes/no judgments about whether personality trait adjectives (e.g., “loving”, “grumpy”, “emotional”) apply to themselves or to a familiar other person, subsequent memory is superior for those traits judged in relation to self than those judged in relation to others [e.g., Klein & Loftus, 1988; Symons & Johnson, 1997]. Likewise, objects that belong to oneself (or are imagined to belong to oneself) are more reliably recalled/recognized than objects that belong to others (an “ownership effect”); in an ownership paradigm, participants observe objects being placed in two locations, and are told that all the items in one location are “owned” by them and all the items in the other location are “owned” by another person. Subsequent memory is reliably superior for self‐owned than other‐owned items [e.g., Cunningham, Turk, MacDonald, & Macrae, 2017].

Self‐reference affects not only memory, however, but also perception. For example, Sui, He, and Humphreys [2012] developed a task in which participants had to make speeded perceptual judgments about whether shape/label pairs matched a previously‐learned contingency. Specifically, participants first learned to associate three simple shapes (triangle, circle, square) with three simple labels that represented self, a familiar other, or an unfamiliar other (e.g., “you”, “mother”, “stranger”). Hence, circle might be associated with “you”, triangle with “mother”, and square with “stranger”. After this brief learning phase, participants completed a series of trials on each of which they were presented with one shape and one label for a short period (100 ms), and made perceptual judgments about whether the shape and label were a match for the learned contingency or a mismatch. Sui et al. found that participants were quicker and more accurate to perceive matches involving the self than they were to perceive matches involving either mother or stranger. Specifically, they found a pattern of accuracy self > mother > stranger and a pattern of response times (RTs) self < mother < stranger on matching trials (but not mismatching trials). This effect, which has been replicated several times [see Sui & Humphreys, 2015], shows that self‐representation influences perception, as well as memory. The importance of studies, such as Sui et al.'s, is captured by Cunningham and Turk [2017, pp. 992–993] when they argue that, “‘New wave’ methodologies, such as Cunningham et al.'s [2017] ownership paradigm and Sui et al.'s [2012] shape association task, have allowed the exploration of the self's influence on cognition to move beyond memory effects to a striking array of automatic self‐processing biases.”

The study of SREs is particularly important when it comes to understanding cognitive processing in various forms of psychopathology that are characterized by atypical self‐representation. If one's self influences or structures cognition, perception, and decision‐making in a fundamental way, then it follows that these facets will be qualitatively different among people with diminished/atypical self‐representation. In this way, difficulties with some aspect of self‐representation might contribute to/underpin core features of a disorder. One developmental disorder that is particularly important to consider in this respect is autism spectrum disorder (ASD).

### Self‐Reference Effects in ASD

Despite a wealth of research into social cognition and awareness of others, in general, among people with ASD, research into self‐awareness in this disorder is relatively sparse. Research into self‐reference effects is beginning to reveal key insights into the nature of self‐experience and self‐representation in this disorder, however. Three studies have converged on the finding that individuals with ASD show a diminished SRE on the traditional trait memory paradigm [Henderson et al., 2009; Lombardo, Barnes, Wheelwright, & Baron‐Cohen, 2007; Toichi et al., 2002]. Moreover, Henderson et al. found that the extent of this diminution was associated significantly with the severity of ASD features (more ASD traits = smaller SRE). Likewise, Grisdale, Lind, Eacott, and Williams [2014, Experiment 2] found that the ownership effect was significantly diminished in adults with ASD, relative to that observed in age‐, IQ‐, and sex‐matched comparison participants. Moreover, Grisdale et al. (Exp. 1) also found that individual differences in the number of ASD traits displayed by a group of neurotypical individuals was associated significantly with the size of the ownership effect, confirming a link between difficulties with social functioning and self‐representation.The usual explanation for these diminished self‐biases in ASD is that people with this disorder have an atypical or impoverished self‐representation, which does not therefore act as an organizational structure to shape encoding of information [see Lind, 2010]. However, an alternative explanation for these findings is that self‐representation is unimpaired in ASD, but somehow “blocked” from influencing memory [because of atypical connectivity between those brain regions underpinning self‐representation and those underpinning memory; see Grisdale et al., 2014]. If this alternative explanation is correct, then people with ASD might well show a typical self‐bias in a domain other than memory. Such a finding would be important for our understanding not only of self‐awareness in ASD, but also psychological functioning more generally in this disorder. As noted above, the importance of self‐awareness for cognition, perception, and decision‐making is increasingly recognized by psychologists, cognitive neuroscientists, and philosophers, so understanding these facets goes hand‐in hand with understanding self‐awareness.^1^

Given the importance of this issue, we investigated it in the current study using one of the “new wave” methodologies that have moved the field on from investigating the effect of self‐reference on memory. Here, we employed the Sui et al. [2012] Shapes Task to investigate for the first time the influence of self‐representation on *perceptual binding*. In Experiment 1, we adopted an individual differences approach to establish whether the size of the self‐bias on the Shapes Task (i.e., the extent of the accuracy and RT advantage for self‐related matches over other‐related matches) was associated with the number of ASD traits [as measured using the Autism‐spectrum Quotient; Baron‐Cohen, Wheelwright, Hill, Raste, & Plumb, 2001] reported by 124 neurotypical individuals.

Given that ASD features are likely to be distributed continuously throughout the general population [e.g., Frazier et al., 2014], studying individual differences in ASD traits and their relation to psychological abilities in the neurotypical population can make an important contribution to our understanding of ASD itself. However, there can still be qualitative differences in the mechanisms/processes that underpin those traits in each population [e.g., Peterson, Wellman, & Liu, 2005; Mandy et al., 2012]. As such, a full understanding requires the study of diagnosed cases, as well as traits in the neurotypical population. Therefore, in Experiment 2, the Shapes Task (as well as the autism‐spectrum quotient and two measures of mindreading) was completed by 22 adults with ASD and 21 age‐, IQ‐, and sex‐matched comparison participants.

## Experiment 1: Method

### Participants

124 students (104 female) from the University of Kent took part in Experiment 1. The average age of participants was 20.02 years (*SD* = 3.22) years. No participant had a history of ASD, according to self‐report. All participants gave informed consent and received course credit in partial fulfillment of their degree, for taking part in the study. The experiment was ethically approved by XXX Psychology Research Ethics Committee.

### Materials and Procedures

***Shapes Task [***Sui et al., 2012 ***]***. Following, participants were first instructed to associate a shape (triangle, square, or circle) to a person label, which could either relate to themselves (“you”), a familiar other (“mother”) or an unfamiliar other (“stranger”). Each shape was associated with a different label (e.g., you = circle, mother = triangle, or stranger = square) and these associations were labeled matches, any alternative combinations were labeled mismatches. The task was to judge if the presented shape and person label was a match or a mismatch.

**Figure 1:**
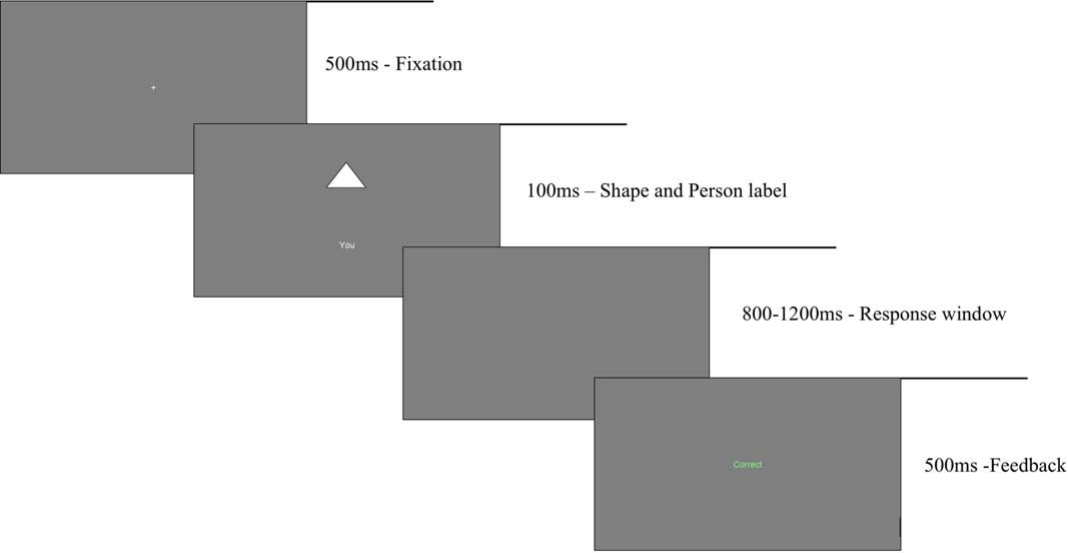
Example of a (correct) trial in the Shapes Task.

In each trial, following a white fixation cross displayed centrally for 500 ms one of the three possible geometric shapes (triangle, square, or circle) was presented centrally above one of the three possible person labels (you, mother or stranger) for 100 ms (see Fig. 1). Participants were then given a variable response window (between 800 and 1200 ms, randomized across trials) to press one of two possible keys, “c” if they judged the combination a match and “m” if they judged it a mismatch. Response feedback followed every trial informing participants of their accuracy. Each participant performed a minimum of 12 training trials prior to 3 blocks of 120 experimental trials. Match and mismatch trials occurred an equal number of times for each person label and the trial order was pseudo‐randomized and presented in a fixed order. Each participant performed one of six versions (counterbalanced across participants) of the task representing each possible shape and person label combination.

The primary dependent measures for the Shapes Task were task accuracy and reaction time (RT). Accuracy for matches was calculated as the proportion of correct responses on match trials for each person label. Likewise, accuracy for mismatches was calculated as the proportion of correct responses on mismatch trials for each person label. We employed accuracy as a DV, rather than another commonly used measure, namely d'. This was because our focus was on the self‐bias for matching judgments specifically (and d' incorporates variance associated with mismatching judgments, as well as matching judgments). However, when d' was employed as the DV in supplementary analyses, the results were substantively identical to when accuracy was employed (see footnotes X and Y).

We also calculated a self‐bias score for matching judgments by subtracting the average combined accuracy for matching judgments about mother and stranger from the average accuracy for matching judgments about self (you). The larger the resulting value, the greater the self‐bias in accuracy for matching judgments. An equivalent self‐bias score was created for mismatching judgments also. We also calculated a self‐bias score for response times on trials in the same manner (combined average RTs of mother and stranger minus you).^2^

#### Autism‐spectrum Quotient (AQ)

The AQ is used widely, and is a valid and reliable measure of ASD traits in people with a full diagnosis and in the general population. Participants read statements (e.g., “I find social situations easy”; “I find myself drawn more strongly to people than to things”) and decide the extent to which each statement applies to them, responding on a 4‐point Likert scale, ranging from “definitely agree” to “definitely disagree”. Scores range from 0 to 50, with higher scores indicating more ASD traits.

### Bayesian Analyses

Bayesian analyses provide an estimation of the relative strength of a finding for one hypothesis over another (i.e., the alternative hypothesis over the null, or vice versa), which allows a more graded interpretation of the data than is possible using *P* values or effect sizes alone [e.g., Dienes, 2014; Rouder, Speckman, Sun, Morey, & Iverson, 2009]. Therefore, we included Bayesian analyses, interpreting results according to Jeffreys [1961] criteria: Bayes factors (BF_10_) > 3 provide firm evidence for the alternative hypothesis (with values > 10, > 30, and >100 providing strong, very strong, and decisive evidence, respectively) and values under 1 provide evidence for the null (with values < 0.33 providing firm evidence). Bayesian analyses were conducted using JASP 0.8.1 (JASP team, 2016).

## Experiment 1: Results

**Figure 2:**
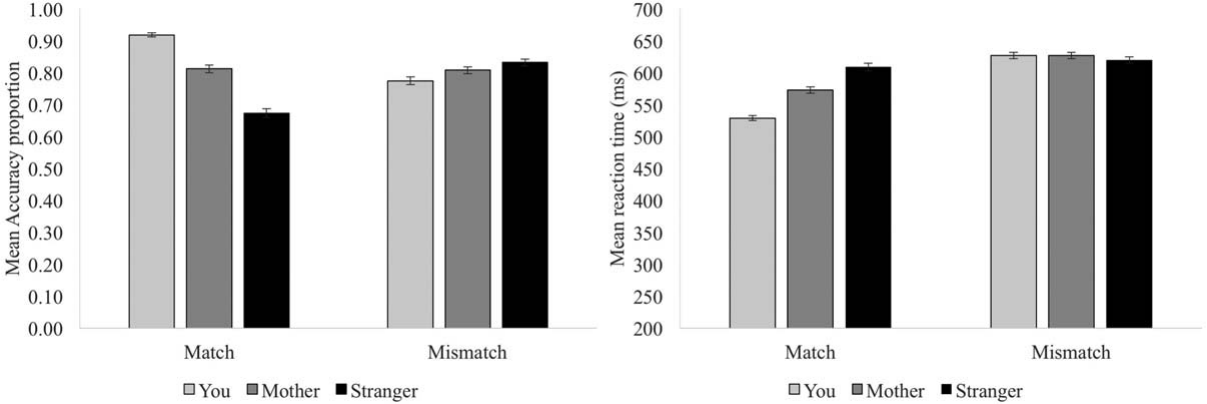
Accuracy and RTs from Experiment 1.

Figure 2 shows the accuracy and RT data in Experiment 1. Two 2 (Trial type: Match/mismatch) × 3 (Person: You/mother/stranger) ANOVAs were conducted, one with accuracy as the dependent variable and the other with RT as the dependent variable. Table 1 shows the results of these ANOVAs, as well as relevant post hoc contrasts/within‐participant t‐tests and the Cohen's *d* associated with each contrast.

**Table 1:** ANOVA Results from Experiment 1

	Effect	*F*	*P*	$\eta_{p}^{2}$	*t*‐tests/contrasts	Cohen's *d* for contrasts
**Accuracy**	Person	74.21	<.001	.38	Self > Mother > Stranger	‐
	Trial type	0.12	.73	.001	Match = mismatch	‐
	Person × Trial type	213.37	.001	.63	**Matching trials** Self > Mother***e	0.97
					Self > Stranger***e	1.70
					Mother > Stranger***e	0.97
					**Mismatching trials** Self < Mother**d	0.30
					Self < Stranger***e	0.54
					Mother < Stranger**d	0.27
**RT**	Person	85.04	.001	.41	Self < Mother < Stranger	
	Trial type	346.16	<.001	.74		
	Person × Trial type				**Matching trials** Self < Mother***e	0.99
					Self < Stranger***e	1.46
					Mother < Stranger***e	0.75
					**Mismatching trials** Self = Mother^a^	0.01
					Self > Stranger*b	0.22
					Mother > Stranger**d	0.26

*P < 0.05; **P < 0.01; ***P < 0.001.

a BF_10_ < 0.33; b BF_10_ = 0.34–0.99; c BF_10_ = 1–2.99; d BF_10_ = 3–99; e BF_10_ > 100.

In sum, for *matching* judgments, accuracy followed a pattern self > mother > stranger (all *p*s < .001, all *d*s >0.97, all BF_10_ > 100). On average, accuracy for matching judgments about self was 18% higher than for matching judgments about mother and stranger (size of the self‐bias = .18, SD = .11). In categorical terms, 119/124 (96%) participants showed a self‐bias in accuracy for matching judgments. Likewise, RT followed a pattern self < mother < stranger (all *p*s < .001, all *d*s >0.75, all BF_10_ > 100). On average, RT for matching judgments about self was 60ms faster than RT for matching judgments about mother and stranger (size of the self‐bias = −0.06s, SD = 0.04). In categorical terms, 112/124 (90%) of participants showed a self‐bias in RT for matching judgments.

In contrast, for *mismatching* judgements, *accuracy* followed a pattern self < mother < stranger (all *p*s < .003, all *d*s >0.27, all BF_10_ > 7.08). Likewise, for mismatching judgments, RT followed a pattern self = mother > stranger (all *p*s < .001, all *d*s >0.75, all BF_10_ > 100). Thus, participants clearly showed a self‐bias in accuracy and RT for matching trials, but not mismatching trials, which replicates findings from Sui et al. [2012].^3^

### Association Analyses

Correlation analyses were conducted to explore the size of the self‐bias for matching judgments in both accuracy and RT on the Shapes Task on the one hand, and score on the AQ, on the other hand. AQ score was non‐significantly associated with both accuracy self‐bias, *r* = .04, *P* = 0.65, BF_10_ = 0.12, and RT self‐bias, *r* = .10, *P* = 0.27, BF_10_ = 0.21.

## Experiment 1: Discussion

In Experiment 1, we replicated precisely the pattern of results on the Shapes Task reported by Sui and colleagues [2012]. Participants were significantly faster and more accurate at making perceptual matching judgments about self than either stranger or mother. Most importantly, however, correlation analyses indicated a negligible and non‐significant association between individual differences in the number of ASD‐like traits (reported using the AQ) and the size of the self‐bias in both accuracy and RT. Bayesian correlation analyses suggested that the data provided moderate to strong evidence in favor of the null hypothesis. These findings contrast with the findings of Grisdale et al. [2014] and Henderson et al. [2009] who report significant associations between number of ASD traits and the size of the self‐bias in memory shown by participants on the ownership and trait memory paradigms, respectively. Given that autism traits did not affect the extent to which self‐reference influenced perception among neurotypical individuals, it seems reasonable to predict that people with a full diagnosis of ASD would show a typical self‐bias on the Shapes task. We tested this prediction in Experiment 2.

## Experiment 2: Method

### Participants

Twenty‐two adults with ASD and 21 neurotypical comparison adults took part. Participant groups were closely matched for age and sex, and as VIQ, PIQ, and FSIQ using the Wechsler Abbreviated Scale for Intelligence‐II [Wechsler, 1999; see Table 1]. Participants in the ASD group had received verified diagnoses, according to conventional criteria [American Psychiatric Association, 2000; World Health Organisation, 1993] and all completed the Autism Diagnostic Observation Schedule [ADOS; Lord et al., 2000].

### Materials and Procedures

Participants from each group completed the Shapes Task and AQ used in Experiment 1. In addition, two mindreading measures were also completed by participants in Experiment 2:

#### *Reading the Mind in the Eyes (RMIE) task [*Baron‐Cohen, Wheelwright, Skinner, Martin, & Clubley, 2001 *]*

The RMIE is a widely used measure of mindreading. Participants were presented with a series of 36 photographs of the eye‐region of the face. On each trial, participants were asked to pick one word from a selection of four to indicate what the person in the picture was thinking/feeling. Scores ranged from a possible 0–36, with higher scores indicating better performance.

#### *Animations Task [e.g*., Abell, Happé, & Frith, 2000 *]*

The task, which is based on Heider and Simmel [1944], required participants to describe interactions between a large red triangle and a small blue triangle, as portrayed in a series of silent video clips. Four clips were apt to invoke an explanation of the triangles' behavior in terms of epistemic mental states, such as belief, intention, and deception. These clips comprise the “mentalizing” condition of the task and were employed in this study.

Each clip was presented to participants on a computer screen. After the clip was finished, participants described what had happened in the clip. An audio recording of participants' responses was made for later transcription. Each transcription was scored on a scale of 0–2 for accuracy (including reference to specific mental states), based on the criteria outlined in Abell et al. [2000]. Twenty percent of transcripts were also scored by two independent raters. Inter‐rater reliability across all clips was excellent according to Cicchetti's [1994] criteria (intra‐class correlation = .85). Accuracy (proportion) among ASD and comparison participants is shown in Table 2.

**Table 2:** Baseline Characteristics among Participants in Experiment 2

	ASD (*n* = 22; 18 male)	NT (*n* = 21; 16 male)	*t*	*P*	*d*
Age	35.84 (11.55)	36.32 (12.01)	−0.13	0.89	0.04
VIQ	101.95 (15.31)	107.00 (9.79)	−1.28	0.21	0.39
PIQ	101.27 (19.85)	106.43 (11.17)	−1.04	0.30	0.32
FSIQ	101.41 (17.21)	107.10 (9.55)	−1.33	0.19	0.41
AQ	31.41 (7.88)	16.19 (5.10)	7.48	<0.001	2.28
ADOS	10.91 (4.17)	‐			
RMIE (proportion)	.63 (.19)	.78 (.10)	−3.29	0.002	1.00
Animations (proportion)	.52 (.28)	.73 (.24)	−2.62	0.012	0.80

## Experiment 2: Results

### Accuracy

**Figure 3:**
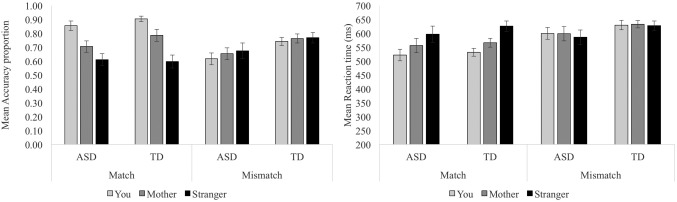
Accuracy and RTs from Experiment 2.

Figure 3 shows the accuracy and RT data in each group in Experiment 2. Two 2 (Group: ASD/neurotypical) × 2 (Trial type: Match/mismatch) × 3 (Person: You/mother/stranger) ANOVAs were conducted, one with accuracy as the dependent variable and the other with RT as the dependent variable. Table 3 shows the results of these ANOVAs, as well as post hoc contrasts/within‐participant *t*‐tests and the Cohen's *d* associated with each relevant contrast.

**Table 3:** ANOVA Results from Experiment 2

	Effect	*F*	*P*	$\eta_{p}^{2}$	*t*‐tests/contrasts	Cohen's *d* for contrasts
**Accuracy**	Person	24.83	< 0.001	.38	Self>Mother>Stranger	‐
	Trial type	3.05	0.09	.07	‐	‐
	Group	2.91	0.10	.07	**‐**	‐
	Person × Trial type	36.49	<0.001	.47	**Matching trials** Self > Mother***^e^	0.87
					Self > Stranger***^e^	1.30
					Mother > Stranger***^e^	0.79
					**Mismatching trials** Self = Mother^b^	0.49
					Self = Stranger^b^	0.51
					Mother = Stranger^a^	0.22
	Group × Trial type	2.37	0.13	.06	‐	‐
	Group × Person	1.57	0.21	.04	‐	‐
	Group × Person × Trial type	0.55	0.58	.01	‐	‐
**RT**	Person	15.42	<0.001	.27	Self<Mother<Stranger	‐
	Trial type	68.58	<0.001	.63	Match < Mismatch	‐
	Group	0.86	0.36	.02	‐	‐
	Person × Trial type	36.95	<0.001	.47	**Matching trials** Self < Mother**^d^	0.47
					Self < Stranger***^e^	1.06
					Mother < Stranger***^e^	1.03
					**Mismatching trials** Self = Mother^a^	0.03
					Self = Stranger^a^	0.17
					Mother = Stranger^b^	0.30
	Group × Trial type	2.93	0.10	.07	‐	‐
	Group × Person	0.79	0.46	.02	‐	‐
	Group × Person × trial type	0.14	0.75	<.01	‐	‐

*P < 0.05; **P < 0.01; ***P < 0.001.

a BF_10_ < 0.33; b BF_10_ = 0.34–0.99; c BF_10_ = 1–2.99; d BF_10_ = 3–99; e BF_10_ > 100.

Just as in Exp. 1, a significant Trial type × person interaction in each ANOVA indicated that both groups of participants were showing a self‐bias in accuracy and RT for matching trials, but not mismatching trials. Crucially, no main or interaction effect involving Group was significant in either ANOVA. Thus, there were no significant differences between the groups in terms of levels or patterns of performance (accuracy or RT) on the Shapes task.

To be clear, accuracy of *matching* judgments about self was significantly greater than either matching judgments about mother or stranger among *both* participants with ASD, all *t*s > 4.43, all *p*s < .001, all *d*s > 0.95, all BF_10_ > 100, and NT participants, *t*s > 3.57, all *p*s < .003, all *d*s > 0.78, all BF_10_ > 20.76. On average, accuracy for matching judgments about self was 20% higher than for matching judgments about mother and stranger among participants with ASD (size of the self‐bias = .20, SD = .16) and 21% higher among NT participants (size of the self‐bias = .21, SD = .16), a between‐group difference that was small and non‐significant, *t*(41) = 0.30, *P* = 0.76, *d* = 0.09, BF_10_ = 0.31. In categorical terms, 19/22 (86%) participants with ASD and 19/21 (90%) of comparison participants showed a self‐bias in accuracy for matching judgments, χ^2^ = 0.18, Fisher's exact *P* = 0.67, *φ* = .06.

Likewise, RTs were faster for *matching* judgments about self than either mother or stranger among both participants with ASD, all *t*s > 2.17, all *p*s < .04, all *d*s > 0.46, all BF_10_ > 3.04, and NT participants, *t*s > 2.12, all *p*s < .05, all *d*s > 0.46, all BF_10_ > 2.81. On average, RT for matching judgments about self was 60ms faster than RT for matching judgments about mother and stranger among participants with ASD (size of the self‐bias = 0.06s, SD = 0.08) and 70ms faster among NT participants (size of the self‐bias = 0.07s, SD = 0.07), a between‐group difference that was small and non‐significant, *t*(42) = 0.44, *P* = 0.66, *d* = 0.13, BF_10_ = 0.33. In categorical terms, 17/22 (77%) participants with ASD and 17/21 (81%) of comparison participants showed a self‐bias in RT for matching judgments, χ^2^ = 0.09, Fisher's exact *P* > 0.99, *φ* = .04.

In contrast, accuracy of *mis*matching judgments about self was non‐significantly different from accuracy of mismatching judgments about mother or stranger among both participants with ASD, all *t*s < 1.32, all *p*s > .20, all *d*s < 0.28, all BF_10_ < 0.48, and NT participants, all *t*s < 0.90, all *p*s > .38, all *d*s < 0.20, all BF_10_ < 0.33. Likewise, RTs were not significantly faster for *mismatching* judgments about self than either judgments about mother or stranger among either participants with ASD, all *t*s < 1.21, all *p*s > .23, all *d*s < 0.26, all BF_10_ < 0.21, or NT participants, all *t*s > 0.49, all *p*s > .63, all *d*s < 0.11, all BF_10_ < 0.25.^4^

### Association Analyses

Correlation analyses were conducted in each group to explore the size of the self‐bias for matching judgments in both accuracy and RT on the Shapes Task, on the one hand, and score on the AQ (and ADOS), on the other hand. Among ASD participants, AQ score was non‐significantly associated with either accuracy self‐bias, *r* = −.10, *P* = 0.67, BF_10_ = 0.29, or RT self‐bias, *r* = −.08, *P* = 0.72, BF_10_ = 0.28. Likewise, ADOS total score was non‐significantly associated with either accuracy self‐bias, *r* < −.01, *P* = 0.99, BF_10_ = 0.26, or RT self‐bias, *r* = −.15, *P* = 0.50, BF_10_ = 0.33. Finally, neither the self‐bias for accuracy or RT was associated with either RMIE performance, *r*s < −.06, *p*s > .79, BF_10_ < 0.27, or Animations task performance, *r*s < .29, *p*s > .19, BF_10_ < 0.58.

Among NT participants, AQ score was non‐significantly associated with either accuracy self‐bias, *r* = .10, *P* = 0.66, BF_10_ = 0.30, or RT self‐bias, *r* = .20, *P* = 0.39, BF_10_ = 0.39. Also, neither the self‐bias for accuracy or RT was associated with RMIE performance, *r*s < .26, *p*s > .25, BF_10_ < 0.50. The association between accuracy and RT self‐biases, and Animations task performance were less easy to interpret, however. The association between RT self‐bias and Animations task performance was moderately positive (the larger the self‐bias, the better the Animation task performance) and close to statistical significance, *r* = .41, *P* = 0.07, BF_10_ = 1.32. The association between accuracy self‐bias and Animations task performance was also moderate in size and close to statistical significance, but this time the association was *negative* (the smaller the self‐bias, the better the Animation task performance), *r* = .39, *P* = 0.07, BF_10_ = 1.13. Note that in both cases, however, the Bayes Factor associated with the associations indicated that the data were inconclusive.

Thus, number of ASD traits/severity of ASD features was non‐significantly related to either accuracy or RT self‐bias in either ASD or comparison participants.

## Experiment 2: Discussion

The results from Experiment 2 dovetailed closely those from Experiment 1; there was no hint of any significant between‐group (ASD/NT) differences in performance on the Shapes Task. Both ASD and closely‐matched NT comparison participants showed an identical pattern of performance across conditions of the Shapes task, showing superior accuracy and faster RTs when making matching judgments about self than about either stranger or mother. The between‐group difference in the size of this self‐bias was associated with a negligible effect size and Bayesian analyses provided moderate‐to‐strong evidence in favor of the null hypothesis with respect to this difference. Moreover, in each group, the size of the self‐bias for matching judgments was equivalent to that shown by the large sample of NT participants in Experiment 1, and the majority of participants in both experiments showed self‐bias for matching judgments. In addition, replicating findings from Experiment 1, number of ASD traits as indexed by score on the AQ (or ADOS among ASD participants only) was not significantly associated with either the accuracy or RT self‐bias for matching judgments in either group of participants; Bayesian analyses again favored the null in every case. Finally, there was no evidence for a reliable association between size of the self‐bias and mindreading ability. Performance on the RMIE task was not significantly associated with either the accuracy or RT self‐bias in either ASD or comparison participants, and the Bayesian analyses favored the null in every case. Performance on the Animations task was moderately associated with accuracy self‐bias and RT self‐bias, but negatively so in the former case and positively so in the latter. These contradictory direction of these associations and the fact that Bayesian analyses indicated that the data were insensitive, as well as the number of comparisons made (inflating the risk of type I error), should lead to a high degree of caution when interpreting these final analyses.

## General Discussion

Findings from experiments 1 and 2 strongly suggest that the extent to which self‐reference influences performance on the Shapes Task is unaffected by ASD or ASD traits. This contrasts with findings from other studies that show the size of the self‐bias on trait memory and ownership paradigms is diminished in people with ASD and predicted by ASD severity/number of ASD traits. What could explain these contrasts?

One explanation is that self‐representation is unimpaired in ASD, but selectively blocked from influencing memory. Certainly, the finding that perceptual binding is enhanced by self‐reference in ASD suggests that at least some aspect of self‐representation is unimpaired in ASD. However, the suggestion that all aspects of self‐representation are unimpaired in ASD and merely blocked from influencing memory is challenged by several other findings. First, individuals with ASD show impairments on some tests of self‐representation that do not involve memory [see Williams, 2010]. Second, some types of self‐bias in memory are undiminished among people with this disorder. Specifically, people with ASD are typical in showing superior memory for their own actions relative to actions they have observed another person make an “enactment effect” [e.g., Baker‐Ward, Hess, & Flannagan, 1990; Engelkamp, 1998]. Grainger, Williams, & Lind, 2014a, 2014b] summarized results across studies and showed that the memory advantage for self‐performed over observed actions was almost identical in (*n* = 239) people with ASD (∼10% advantage) and (*n* = 240) comparison individuals (∼11% advantage).

The explanation for this range of findings across studies might lie in the type/level of self‐representation that underpins the self‐bias on each of these different paradigms. In the trait memory paradigm, participants make judgments about whether trait words apply to them (“Does ‘cheerful’ apply to *me*?”), which clearly requires the formation of a second‐order representation of oneself. Thus, at the point of encoding the relevant information in the trait memory paradigm, the self is the *object* of thought (the “me” in James' terms). In the ownership paradigm, participants arguably have to make a judgment about the owned (or non‐owned) status of objects at the point of encoding (“Is this object *mine*?”). In contrast, in both the shapes and enactment paradigms, no such second‐order representation is required. In the Shapes Task, participants have to make a speeded perceptual judgment about whether or not shape‐label pairs match a learned contingency. In this case, we suggest that a first‐order representation of self (i.e., the self as the *subject* of thought; the “I”, in James' terms) is all that is required to bias accuracy and RT in favor of self‐relevant matches. No second‐order reflection of the form, “Does ‘triangle’ match *me*?”, is required to bias such perceptual judgments, we suggest. Likewise, in traditional enactment paradigms, a participant performs a series of actions and also observes another person performing a distinct set of actions, after which memory for the actions is tested. There is no obvious sense in which any participant capable of basic action‐monitoring needs to form a second‐order representation about whether or not they are performing the action. Again, the subjective self is all that is required to bias information encoding in such circumstances [Grainger, Williams, & Lind, 2016a, 2016b]. This suggestion is consistent with evidence from neuroimaging studies that distinguish subjective from objective aspects of self. For example, Schmitz and Johnson [2007] suggest that the ventro medial prefrontal cortex (vmPFC) underpins pre‐reflective orientating to/decisions about self‐relevant stimuli, whereas the dorsal medial prefrontal cortex (dmPFC) contributes to conscious reflection on and evaluation of oneself. In this context, it is important to note that the dmPFC has been implicated in the self‐bias on both the trait memory and ownership paradigms [Sui and Humphreys 2017; Turk, Van Bussel, Waiter, & Macrae, 2011], but not the Shapes paradigm which relies instead on vmPFC activation [see Sui & Humphreys, 2015]. Moreover, this distinction between ventral and medial sections of the PFC seems consistent with another important “dissociation” in the current article, namely between self‐reference and mindreading. In Experiment 2, participants with ASD showed characteristic significant impairments on two measures of mindreading, despite showing the typical SRE on the Shapes Task. Importantly, the dmPFC is a core component of the network of brain regions that underpins mindreading [e.g., Isoda & Noritake, 2013] and, early in life, supports specifically the kind of triadic joint attention that is an early manifestation of (or perhaps precursor to) mindreading [e.g., Grossman & Johnson, 2007; Mundy, 2009], all of which this is consistent with the argument that only certain forms of self‐reference are linked to social‐cognitive abilities more generally.

If our interpretation is correct, then this suggests that only second‐order representations of self (the “me”) are atypical/impoverished among people with ASD. Certainly, this is in keeping with findings that metacognitive monitoring (i.e., diminished second‐order/meta‐representation of one's cognitive activity) is impaired in ASD [e.g., Grainger et al., 2014a, b, Williams, Bergström, & Grainger, 2016]. However, an alternative possibility (suggested by an anonymous reviewer) is that participants with ASD showed an undiminished self‐bias on the Shapes Task because the stimuli was devoid of any emotional content. On the traditional trait memory paradigm, described above, participants make judgments about whether emotionally‐valanced adjectives apply to themselves or others, whereas the label‐shape associations in the Shapes Task appear emotionally‐neutral. Given this, it may be that the diminished self‐reference effect on the trait memory paradigm (and ownership paradigm), but undiminished self‐reference effect on the Shapes Task (and action monitoring tasks), reflects the different emotional demands inherent to each type of task.

These two possibilities could be tested directly by (among other means) comparing performance of individuals with ASD on a test of self‐referential memory that requires *explicit* judgments to be made about self with a test in which encoding of self‐related information is only *implicit* or incidental to the task at hand [e.g., Cunningham et al., 2014]. If only second‐order self‐representations are diminished in ASD, then ASD‐specific impairments should be expected on the explicit evaluative task only. In contrast, if the root of the diminished self‐reference effects in ASD is a difficulty with emotion‐processing, then even the self‐bias should be diminished among people with ASD on both the incidental/implicit and explicit versions of the trait memory task, because both involve the self‐referential processing of emotionally‐valanced adjectives. Regardless, the current results suggest strongly that perceptual binding is supported by self‐reference in ASD just as it is in typical development. These findings inform theories of perception, as well as self‐awareness, in ASD, and suggest an important dissociation between self‐referential processing and social cognition more generally.

## Footnotes

1 Studies of self‐referencing have also found that neurotypical individuals show a positivity bias, endorsing more positive traits about themselves than negative traits [e.g., Pfeifer et al., 2013], but that this bias is also diminished in ASD [Burrows et al., 2017].

2 The size of the self‐bias was determined by subtracting average performance (for both accuracy and RT) *across* the mother *and* stranger conditions from performance in the self condition. An alternative approach would have been to create two types of self‐bias score [as Lombardo et al., 2007, did]. For both accuracy and RT, self‐bias could have been indexed by calculating self minus mother, on the one hand (self‐bias 1), and self minus stranger, on the other hand (self‐bias 2). The disadvantage to this approach would be that it increases the number of statistical comparison necessary from 12 to 24, which would have increased the risk of making Type I errors. Nonetheless, after a suggestion from an anonymous reviewer, we conducted the additional analyses post hoc. These revealed that—in keeping with our original association analyses reported above—in Experiment 1, AQ was non‐significantly associated with either self‐bias 1 for accuracy or RT (*r*s < .04, *p*s > .64), or self‐bias 2 for accuracy or RT (*r*s < .13, *p*s > .17). Likewise, in Experiment 2, AQ was non‐significantly associated with either a) self‐bias 1 for accuracy or RT in either ASD (*r*s < −.14, *p*s > .53) or comparison participants (*r*s < .29, *p*s > .21), or b) self‐bias 2 for accuracy or RT in either ASD (*r*s < −.14, *p*s > .52) or comparison participants (*r*s < .27, *p*s > .23). Thus, no matter which way the self‐bias score is calculated, we found no evidence that it was associated with ASD features or traits.

3 There were no systematic differences between males and females in terms of accuracy or RT on the Shapes task. The two ANOVAs reported for Exp. 1 were reconducted but each including a sex variable. None of the main or interaction effects involving Sex were significant or associated with anything other than a small effect size, all *F*s < 1.95, all *p*s > .14, all $\eta_{p}^{2}<\;.02.$ Thus, there were no significant differences between males and females in terms of either overall level or patterns of performance on the Shapes task.

4 Using d', rather than accuracy, produces the same results as the proportion accuracy scores employed in Experiment 2. A 2 (Group: ASD/neurotypical) × 3 (Person: You/mother/stranger) mixed ANOVA on d' scores yielded a significant main effect of Person, *F*(2, 82) = 24.39, *P* < 0.001, $\eta_{p}^{2}=$ .37, reflecting a pattern of d' self > d' mother > d' stranger among both groups of participants (all ts > 4.06, all *ps* <.002, all *ds* >.53, all BF10 >26.07). Neither the main effect of Group, *F*(1, 41) = 3.05, *P* = 0.17, $\eta_{p}^{2}=$ .05, nor the Group × person interaction effect was significant, *F*(2, 82) = 1.64, *P* = 0.20, $\eta_{p}^{2}=$ .04. The size of the self‐bias in *d*' scores was 0.66 (SD = 0.61) among participants with ASD and 0.70 (SD = 0.99) among NT participants, a difference between the groups that was small and non‐significant, *t*(41) = 0.13, *P* = 0.90, *d* = 0.04, BF_10_ = 0.30.
